# Smoking cessation and survival among people diagnosed with non-metastatic cancer

**DOI:** 10.1186/s12885-020-07213-5

**Published:** 2020-08-05

**Authors:** Tracey E. Barnett, Yan Lu, Aaron W. Gehr, Bassam Ghabach, Rohit P. Ojha

**Affiliations:** 1 School of Public Health, University of North Texas Health Science Center, 3500 Camp Bowie Blvd., Fort Worth, TX 76107 USA; 2 Center for Outcomes Research, JPS Health Network, 1500 S. Main Street, Fort Worth, TX 76104 USA; 3 JPS Oncology and Infusion Center, JPS Health Network, 610 W. Terrell Ave., Fort Worth, TX 76104 USA; 4 Department of Biostatistics and Epidemiology, University of North Texas Health Science Center, 3500 Camp Bowie Blvd., Fort Worth, TX 76107 USA

**Keywords:** Cancer, Smoking, Cessation, Survival

## Abstract

**Background:**

We aimed to estimate the effects of smoking cessation on survival among people diagnosed with cancer.

**Methods:**

We used data from a Comprehensive Community Cancer Program that is part of a large urban safety-net hospital system. Eligible patients were diagnosed with primary invasive solid tumors between 2013 and 2015, and were current smokers at time of diagnosis. Our exposure of interest was initiation of smoking cessation within 6 months of cancer diagnosis. We estimated inverse probability weighted restricted mean survival time (RMST) differences and risk ratio (RR) for all cause 3-year mortality.

**Results:**

Our study population comprised 369 patients, of whom 42% were aged < 55 years, 59% were male, 44% were racial/ethnic minorities, and 59% were uninsured. The 3-year RMST was 1.8 (95% CL: − 1.5, 5.1) months longer for individuals who initiated smoking cessation within 6 months of cancer diagnosis. The point estimate for risk of 3-year mortality was lower for initiation of smoking cessation within 6 months of diagnosis compared with no initiation within 6 months (RR = 0.72, 95% CL: 0.37, 1.4).

**Conclusions:**

Our point estimates suggest longer 3-year survival, but the results are compatible with 1.5 month shorter or 5.1 longer 3-year overall survival after smoking cessation within 6 months of cancer diagnosis. Future studies with larger sample sizes that test the comparative effectiveness of different smoking cessation strategies are needed for more detailed evidence to inform decision-making about the effect of smoking cessation on survival among cancer patients.

**Implications for Cancer survivors:**

The benefits of smoking cessation after cancer diagnosis may include longer survival, but the magnitude of benefit is unclear.

## Background

Smoking is associated with adverse outcomes including mortality for people diagnosed with cancer [1–6]. The National Comprehensive Cancer Network Clinical Practice Guidelines thus recommends smoking cessation for patients diagnosed with cancer at any stage [7], but 50–83% continue smoking after cancer diagnosis [8]. Despite certain substantiated benefits of smoking cessation [6], insufficient direct evidence is available about whether smoking cessation improves survival among people diagnosed with cancer [2, 9–11]. For example, a 2019 Cochrane Systematic Review [12] did not identify any randomized controlled trials (RCTs) that reported the effect of smoking cessation interventions on lung cancer survival. Particularly informative would be evidence related to the magnitude of survival benefit related to early smoking cessation. Nevertheless, an adequately powered RCT with sufficient longitudinal follow-up to assess the effects of smoking cessation on survival may be infeasible. Alternate approaches should be explored to address this gap in the evidence.

The target trial framework is a recent advancement to facilitate addressing questions using observational data that may be infeasible through RCTs [13]. This framework uses strengths of RCTs to guide the design of observational studies. In particular, the target trial framework can help reduce the effect of biases from misaligned time zero, which can often be more severe than unmeasured confounding [13–16]. Prior studies that used this framework reported similar estimates between observational studies and RCTs that assessed the effect of antiretroviral treatments on HIV outcomes [17], or the effect of statins on disease prevention [18]. In the absence of an RCT, this approach could help generate useful evidence about the effects of smoking cessation among people diagnosed with cancer. Therefore, we aimed to assess the effect of smoking cessation on survival among people diagnosed with cancer using a target trial framework.

## Methods

### Setting

The JPS Oncology and Infusion Center is a Comprehensive Community Cancer Program that has been accredited by the Commission on Cancer since 2010. The Oncology and Infusion Center is part of JPS Health Network, a large urban safety-net system that serves Tarrant County, TX. The network is a primary source of care for underserved and vulnerable populations, which includes individuals who are uninsured, under-insured, racial/ethnic minorities, immigrants, disabled, homeless, and inmates [19–21].

### Data sources and eligibility criteria

We linked data between the JPS Oncology and Infusion Center Registry and electronic health records from the entire JPS Health Network, which allowed for ascertaining additional health information documented during interactions with the healthcare system prior to and following cancer diagnosis. Patients eligible for our analyses were aged ≥18 years, diagnosed with a first primary invasive solid tumor between 2013 and 2015, received all or part of the first course treatment at JPS Oncology and Infusion Center, and were current smokers at the time of cancer diagnosis (i.e., on or within 30 days before the date of cancer diagnosis). We excluded patients with metastatic cancers because smoking cessation may not be a priority when life expectancy is limited.

### Outcomes

Our primary outcome of interest was 3-year all-cause mortality. Vital status was ascertained through the institutional registry from JPS Oncology and Infusion Center. The cancer registrars routinely check multiple sources of mortality information (e.g., Bureau of Vital Statistics, Social Security Death Index, Texas Obituaries, Fort Worth Star Telegram Obituaries, Tarrant County Medical Examiner, JPS electronic medical records, etc.) to maintain at least 90% follow-up at 5 years for Commission on Cancer accreditation.

### Intervention and follow-up

We defined intervention as initiation of smoking cessation within 6 months of cancer diagnosis, which was based on the date of provider-documented change in smoking status from current to former. Baseline (i.e., time zero) for the first trial was thus the date of cancer diagnosis and patients were followed until death, loss to follow-up, or end of study, whichever occurred first. Patients were classified as initiating the intervention if smoking cessation was documented within the first 30 days after cancer diagnosis and not initiating the intervention if smoking cessation was not documented within the first 30 days after cancer diagnosis. We sequentially applied the eligibility criteria and intervention definition for the 5 successive 30-day intervals (i.e., through 180 days after cancer diagnosis) to generate a total of 6 trials, where baseline for each trial was the beginning of each 30-day interval. Consequently, patients could have been eligible for up to 6 trials but were no longer eligible for subsequent trials if smoking cessation was initiated in a previous trial. We did not have sufficient information to accurately determine duration of smoking cessation, which precluded an adherence-adjusted (i.e., per protocol) analysis.

### Data analysis

We pooled data from all 6 sequential trials and used a marginal structural model [22] with pseudo-observations [23, 24] to estimate risk ratio (RR) and restricted mean survival time (RMST) difference for 3-year mortality. The estimates generated from this approach are analogous to an intention to treat estimate from a pragmatic trial [13]. The pooling of sequential trials also allows for reducing variance when few initiators of the intervention (smoking cessation in our study) are observed. In addition, a critical advantage of the pseudo-observation approach is that the estimates are not sensitive to the assumption of proportional hazards during follow-up, as in Cox proportional hazard regression [25]. RRs and RMST differences are also more easily interpreted by patients and providers than hazard ratios, which could facilitate shared decision-making [25–28].

Specifically, we fit a logistic regression model to compute stabilized inverse probability weights [29] (mean = 1.0, range = 0.29–4.4) of smoking cessation within 6 months. These weights were conditioned on a minimal sufficient set of covariates to reduce confounding bias identified by applying the back-door criterion to a directed acyclic graph of dependencies between smoking cessation and mortality (Supplementary Figure S1). The stabilized inverse probability weights were thus conditioned on baseline measurements of age (18–44, 45–54, 55–64, or > 65 years), gender, race/ethnicity (non-Hispanic White, non-Hispanic Black, Hispanic, and Other), cancer type (smoking- and non-smoking-related cancers [30]), cancer stage (Surveillance, Epidemiology, and End Results summary stage categories of localized or regional), packs of cigarettes per day (≤1 or > 1 packs per day), alcohol use (current and formal user, or never user), insurance coverage (uninsured, public insurance, private insurance, and other insurance), comorbidity (NCI Comorbidity Index [31] score categorized as 0, or > 0), BMI (BMI < 25, 25 ≤ BMI < 30, or BMI ≥ 30), and marital status (single/never married, married, separated/divorced/widowed). We applied these weights in a generalized linear model with pseudo-observations for the survival function at 3 years and log link to estimate RRs comparing initiation and non-initiation of smoking cessation within 6 months to no smoking cessation within 6 months. In addition, we applied these weights in a generalized linear model with pseudo-observations for restricted mean survival function at 3 years to estimate RMST differences between initiation and non-initiation of smoking cessation within 6 months. RMST provides an absolute measure of differences in survival time at the specified time horizon. For example, an estimate of 1.5 when comparing initiation and non-initiation of smoking cessation at a 3-year time horizon would be interpreted as a 1.5 month longer mean survival after smoking cessation at 3-years of follow-up. We estimated 95% compatibility limits (CL) [32] for RR and RMST difference based on clustered standard errors to account for individuals who contributed information to multiple trials. We lacked sufficient sample size to explore effects by cancer type.

### Sensitivity analysis

Unmeasured confounding is a threat to the validity of observational studies. Consequently, we explored the sensitivity of our estimates to unmeasured confounding by computing E-values [33]. The E-value represents the required effect of the unmeasured confounder (or matrix of confounders) on both smoking cessation within 6 months and mortality, conditional on measured covariates, to nullify our estimates.

## Results

**Fig. 1:**
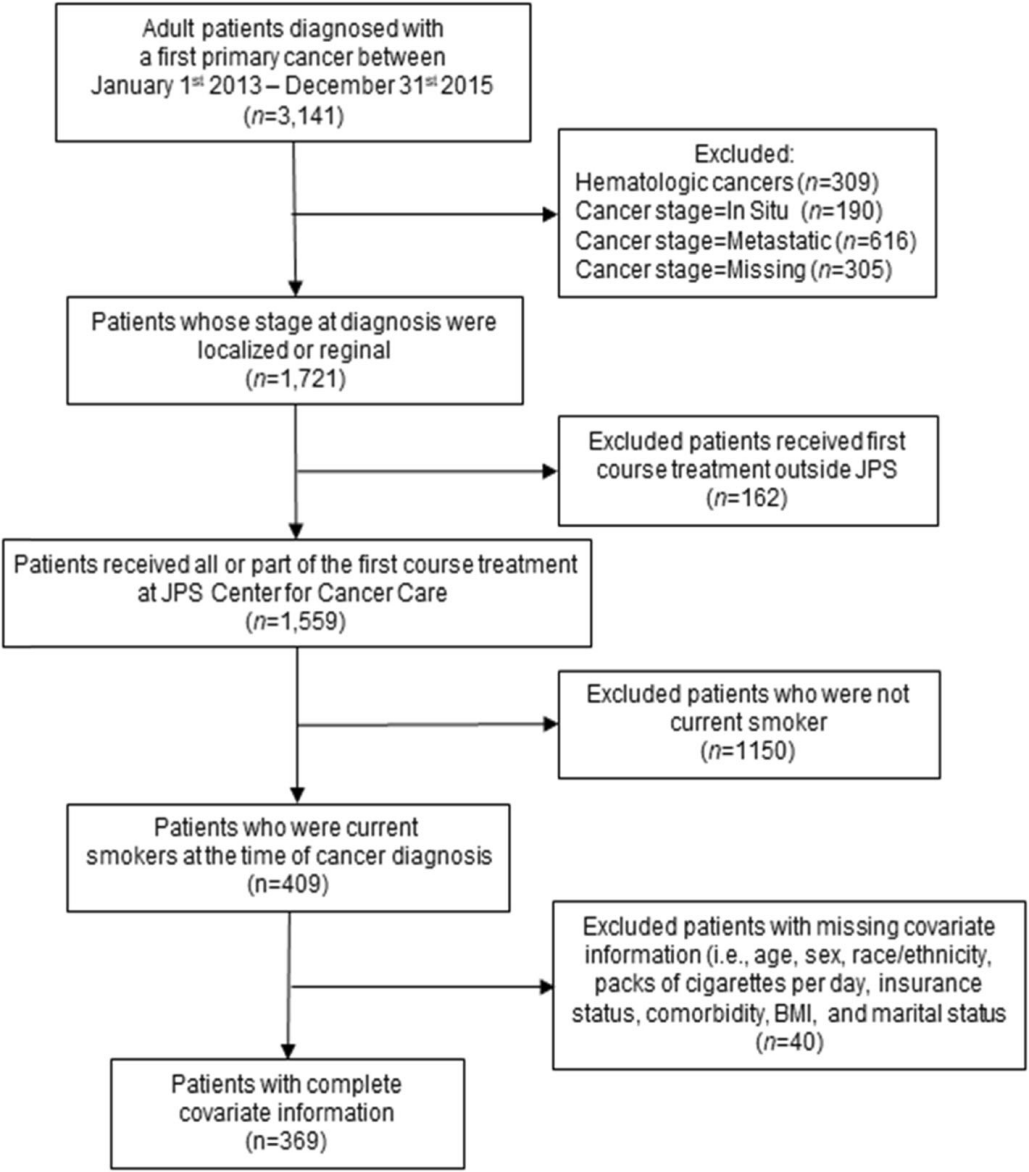
Selection of cancer patients who were smokers at the time of cancer diagnosis

Our eligible population comprised 409 cancer patients, of whom 369 patients with complete covariate information were included in our analysis (Fig. 1). Evaluable patients contributed 1654 observations after pooling the 6 sequential trials (Supplementary Table S2). Table 1 summarizes the characteristics of the study population. Briefly, 78% were aged 45–64 years old, 59% were male, 44% were racial/ethnic minorities, 59% were uninsured, and 57% were diagnosed with localized stage cancers. In addition, 51% smoked more than one pack of cigarettes per day at the time of diagnosis, 31% initiated smoking cessation within 6 months of diagnosis, and 22% died by the end of 3-year follow-up after cancer diagnosis. The median follow-up time was 15 months (interquartile range: 5.8–22 months).

**Table 1:** Characteristics of adult cancer patients who were diagnosed with a first primary invasive non-metastatic solid malignancy between 2013 and 2015 and were smokers at baseline

Characteristics	All patients (*n* = 369) *n* (%)	Patients who quit within 6 months of diagnosis (*n* = 116) *n* (%)	Patients who did not quit within 6 months of diagnosis (*n* = 253) *n* (%)
Age			
18–44 years	50 (14)	15 (13)	35 (14)
45–54 years	107 (29)	30 (26)	77 (30)
55–64 years	179 (49)	60 (52)	119 (47)
65 years and up	33 (8.9)	11 (9.5)	22 (8.7)
Sex			
Male	216 (59)	71 (61)	145 (57)
Female	153 (41)	45 (39)	108 (43)
Race/Ethnicity			
Non-Hispanic White	206 (56)	60 (52)	146 (58)
Non-Hispanic Black	111 (30)	33 (28)	78 (31)
Hispanic	44 (12)	19 (16)	25 (9.9)
Non-Hispanic other	8 (2.2)	4 (3.5)	4 (1.6)
Insurance coverage			
Uninsured	217 (59)	75 (65)	142 (56)
Public insurance	121 (33)	7 (6.0)	90 (36)
Private insurance	23 (6.2)	31 (27)	16 (6.3)
Other insurance^a^	8 (2.2)	3 (2.6)	5 (2.0)
Marital status			
Single	166 (45)	61 (53)	105 (42)
Married	104 (28)	31 (27)	73 (29)
Separated/Divorced/Widowed	99 (27)	24 (21)	75 (30)
Smoke intensity			
> 1 pack of cigarettes per day	189 (51)	54 (47)	135 (53)
≤ 1 pack of cigarettes per day	180 (49)	62 (53)	118 (47)
Alcohol consumption			
Never drinker	205 (56)	70 (60)	135 (53)
Current or former drinker	164 (44)	46 (40)	118 (47)
Body Mass Index (BMI)			
BMI < 25	133 (36)	47 (41)	86 (34)
25 ≤ BMI < 30	96 (26)	22 (19)	74 (29)
BMI ≥ 30	140 (38)	47 (41)	93 (37)
NCI comorbidity index score			
0	227 (62)	71 (61)	156 (62)
> 0	142 (38)	45 (39)	97 (38)
SEER summary stage			
Local	210 (57)	60 (52)	150 (59)
Regional	159 (43)	56 (48)	103 (41)
Cancer type			
Smoking-related cancers (total)	230 (62)	82 (71)	148 (58)
Lung/Bronchus/Trachea	46 (12)	16 (14)	30 (12)
Colorectal	40 (11)	19 (16)	21 (8.3)
Oral/Pharynx/Larynx	38 (10)	24 (21)	14 (5.5)
Kidney/Ureter/Bladder	37 (10)	9 (7.8)	28 (11)
Liver	33 (8.9)	5 (4.3)	28 (11)
Pancreas	13 (3.5)	3 (2.6)	10 (4.0)
Cervix uteri	12 (3.3)	1 (0.86)	11 (4.3)
Esophagus/Stomach	11 (3.0)	5 (4.3)	6 (2.4)
Cancers not related to smoking (total)	139 (38)	34 (29)	105 (42)
Breast	30 (8.1)	12 (10)	18 (7.1)
Prostate	24 (6.5)	3 (2.6)	21 (8.3)
Other	85 (23)	19 (16)	66 (26)
Mortality by 3 years after diagnosis	82 (22)	23 (20)	59 (23)
Total follow-up time (person-years)	428	153	275

^a^Other insurance included Tricare, Military, Veteran’s affair, and Indian/Public health services

**Fig. 2:**
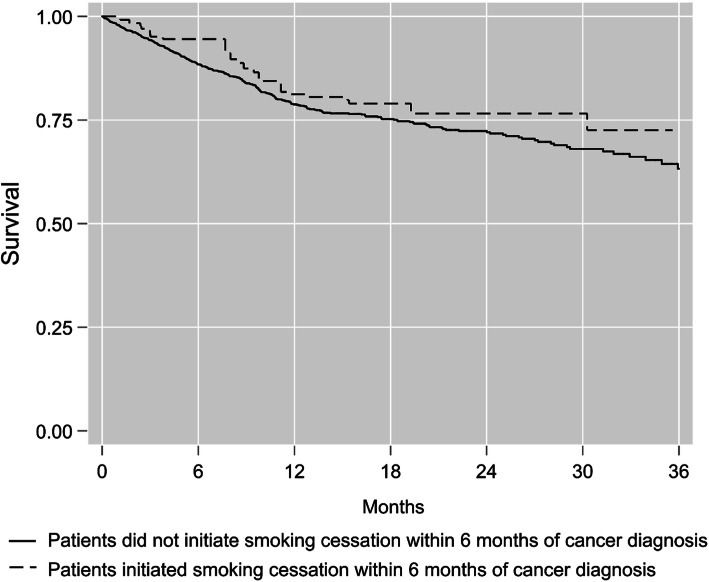
Marginal survival curves for cancer patients who initiated or did not initiate smoking cessation within 6 months of cancer diagnosis

**Fig. 3:**
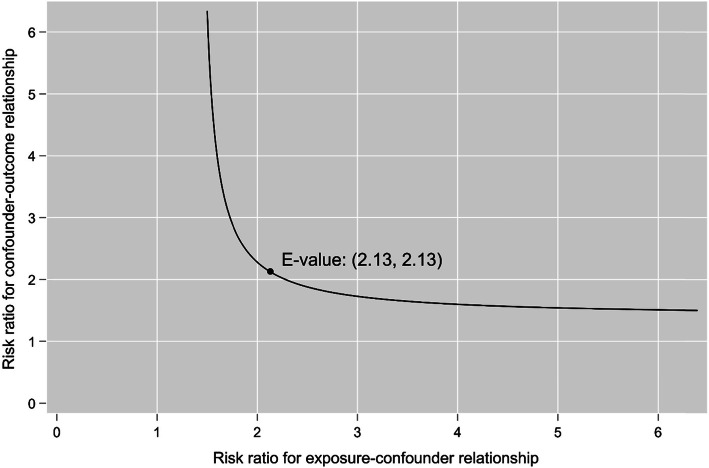
E-value for the effect of smoking cessation within 6 months of cancer diagnosis on 3-year mortality among cancer patients

Figure 2 illustrates the marginal survival curves for patients who initiated or did not initiate smoking cessation within 6 months of cancer diagnosis. Given 3 years follow-up, the average survival time was 1.8 (95% CL: − 1.5, 5.1 months) months longer for individuals who initiated smoking cessation within 6 months of cancer diagnosis (Table 2). Table 2 also summarizes the effect of initiating smoking cessation within 6 months of cancer diagnosis on 3-year mortality. The risk of 3-year all-cause mortality was 28% lower relative to no initiation of smoking cessation within 6 months of cancer diagnosis, but our estimates could be compatible with up to a 63% lower risk or 40% higher risk (RR = 0.72; 95% CL: 0.37, 1.4). Figure 3 illustrates the range of estimates for unmeasured confounding that could explain our results. The E-value for the point estimate was RR = 2.1.

**Table 2:** Survival outcomes among cancer patients who initiated or did not initiate smoking cessation within 6 months of cancer diagnosis

	Estimate	95% CL^a^
Restricted mean survival time given 3-year follow-up		
Initiated smoking cessation	30 months	26, 33 months
Did not initiate smoking cessation	28 months	26, 30 months
Difference in RMST^b^ between initiators and non-initiators	1.8 months	−1.5, 5.1 months
Mortality risk by the end of 3-year follow-up		
Initiated smoking cessation	0.26	0.097, 0.43
Did not initiate smoking cessation	0.37	0.25, 0.48
Mortality risk ratio of quitters vs. non-quitters	0.72	0.37, 1.4

^a^Compatibility limits

^b^Restricted mean survival time

## Discussion

Smoking cessation is considered beneficial for general health, but the magnitude of survival benefits for people diagnosed with cancer is unclear. In particular, the interval of smoking cessation after cancer diagnosis is important considering potentially long smoking histories among people diagnosed with cancer [34]. Our point estimates suggest longer survival and lower 3-year mortality after smoking cessation within 6 months of cancer diagnosis compared with no smoking cessation within 6 months, but this estimate is imprecise and compatible with strong beneficial effects or modest harmful effects. For example, smoking cessation within 6 months of cancer diagnosis prolonged mean survival by 1.8 months after 3 years of follow-up, but this estimate was compatible with 1.5 months shorter or 5.1 months longer survival compared with no smoking cessation within 6 months of cancer diagnosis.

Several limitations should be considered when interpreting our results. A key assumption of inference to consider in our analyses is exchangeability (i.e. no confounding) [35]. We adjusted for key measured pre-exposure common causes of smoking cessation and mortality, but bias from unmeasured confounding is possible. For example, psychological distress may affect smoking cessation and mortality, but information about psychological distress was not uniformly available. We estimated the E-value [33] to assess the robustness of the point estimate to unmeasured confounding. The E-value was 2.1, which indicates that the risk ratio for the unmeasured covariate(s) in relation to both the exposure and outcome must be at least 2.1 to nullify the mortality reduction indicated by the point estimate after smoking cessation. Despite plausibility, we question whether this magnitude of unmeasured confounding is possible given the extent of covariate adjustment. In addition, alignment of time zero, based on the design of our study, can further attenuate the effect of confounding [13–16].

Another key assumption for inference is consistency [36], which requires unambiguous definition of the exposure. We defined the period during which smoking cessation occurred, but we lacked sufficient information to define precisely how smoking cessation was achieved. For example, we are unclear whether patients used nicotine replacement therapy, counseling, or other interventions. Our estimates thus represent the maximum potential effect of smoking cessation but not necessarily a single intervention. In addition, our estimates are analogous to an intention to treat effect. Differences in adherence to smoking cessation could result in over- or underestimation of effect (i.e. per protocol effect), but inconsistent post-smoking cessation data precluded estimating an adherence-adjusted estimate.

Prior studies [37–42] did not address the effect of smoking cessation within a well-defined interval after cancer diagnosis, which could be useful for informing decisions about early smoking cessation. For example, one study [38] assessed the effect of recent cessation on overall survival among lung cancer patients who were treated with surgery. Recent cessation was defined as any time between cancer diagnosis and surgery, but the duration between diagnosis and surgery was not specified. More importantly, the results of prior studies are difficult to interpret as possible effects of smoking cessation because of misaligned time zero. Unlike RCTs, where time zero is well-defined, observational studies are sensitive to systematic errors that occur because of misaligned time zero. This misalignment occurs when eligibility, treatment (exposure) assignment, and follow-up do not occur simultaneously [14]. In particular, some studies used prevalent exposures, where smoking cessation occurred prior to eligibility or follow-up, which resulted in truncation of person-time contributed to the study [38, 41]. Other studies assigned individuals as having quit smoking after eligibility and start of follow-up, which resulted in immortal time bias [37, 39, 40, 42]. The consequence can be severe bias away from the null. For example, one study [38] reported that the mortality hazard was 0.34 (95% CL: 0.16, 0.71) times lower for recent cessation compared with continued smoking, which suggests more extreme effects on mortality than the 3-year mortality risk ratio of 0.72 (95% CL: 0.37, 1.4) observed in our study. Such overestimation related to misaligned time zero has been reported in other contexts [18]. In addition, duration of follow-up, frequency of smoking cessation, and population characteristics could contribute to differences between our study and prior studies.

Promotion of smoking cessation is an important part of a comprehensive treatment plan for cancer patients [43]. Despite the known benefits of cessation and the known risks of continuing to smoke, cancer diagnosis is underused [44] and undervalued [43] as an opportunity to promote cessation. Clinicians may have concerns that a cancer diagnosis is an inopportune time to discuss smoking because the patient is overwhelmed with significant life changes having received bad news and must manage and adhere to a new treatment regimen. Nevertheless, properly framed, a cancer diagnosis could be an educational opportunity to discuss the potential for improved outcomes with smoking cessation and support. Patients report a willingness to attempt cessation. For example, a recent study reported that cancer survivors who continued to smoke remained motivated to quit even beyond the initial diagnosis [45].

People diagnosed with cancer may have several barriers to smoking cessation. Cancer diagnosis generally occurs at older ages and people diagnosed with cancer may have a long history of smoking. The combination of many years of addiction and psychological distress from a cancer diagnosis can make smoking cessation challenging [46, 47]. Additionally, vulnerable populations, such as socioeconomically disadvantaged individuals, have a higher frequency of smoking and tobacco use [6], and have smoked for more years than those living above the poverty rate [48]. Such populations often rely on safety-net institutions for healthcare, including cancer treatment [21]. Despite higher frequency and heavier [49] smoking among underserved patients, safety-net institutions may have less resources to provide effective smoking cessation strategies such as groups and therapies.

## Conclusions

In summary, smoking cessation is recommended for cancer survivors at any time during survivorship by the National Comprehensive Cancer Network [7], but unknown is the magnitude of benefit of early smoking cessation. Our results suggest that smoking cessation within 6 months of cancer diagnosis may increase survival, but the magnitude of effect is imprecise, which precludes definitive conclusions. Our results strengthen the available evidence by addressing limitations of prior analyses and improve understanding the potential interventional effects of early smoking cessation on a population level. Nevertheless, future studies with larger sample sizes that test the comparative effectiveness of different smoking cessation strategies are needed to provide more detailed evidence to inform decision-making. In addition, future studies should consider potential individual and structural barriers to implementing smoking cessation strategies, particularly in vulnerable populations.

## Supplementary information

**Additional file 1: Supplementary Figure S1.** Directed acyclic graph illustrating dependency assumptions for the effect of smoking cessation on cancer mortality. **Supplementary Table S2.** Distribution of observations related to eligibility changes across sequential trials.

## Data Availability

The data analyzed for the current study are not publicly available to protect patient confidentiality but are available on reasonable request to the corresponding author and review by the JPS Health Network External Data Governance Committee.

## Abbreviations

RMST: Restricted mean survival time
RR: Risk ratio
RCT: Randomized controlled trials
JPS: John Peter Smith (hospital name)
BMI: Body mass index
