# Supplementary material for: Smoking cessation and survival among people diagnosed with non-metastatic cancer
Source: BMC Cancer. 2020 Aug 5;20:726. doi: 10.1186/s12885-020-07213-5 (PMC7405359; doi:10.1186/s12885-020-07213-5)
Supplement: Supplementary file 1 — Additional file 1: Supplementary Figure S1. Directed acyclic graph illustrating dependency assumptions for the effect of smoking cessation on cancer mortality. Supplementary Table S2. Distribution of observations related to eligibility changes across sequential trials. [file 12885_2020_7213_MOESM1_ESM.docx]

**SUPPLEMENTARY MATERIALS**

**Supplementary Figure S1. Directed acyclic graph illustrating dependency assumptions for the effect of smoking cessation on cancer mortality.**

**
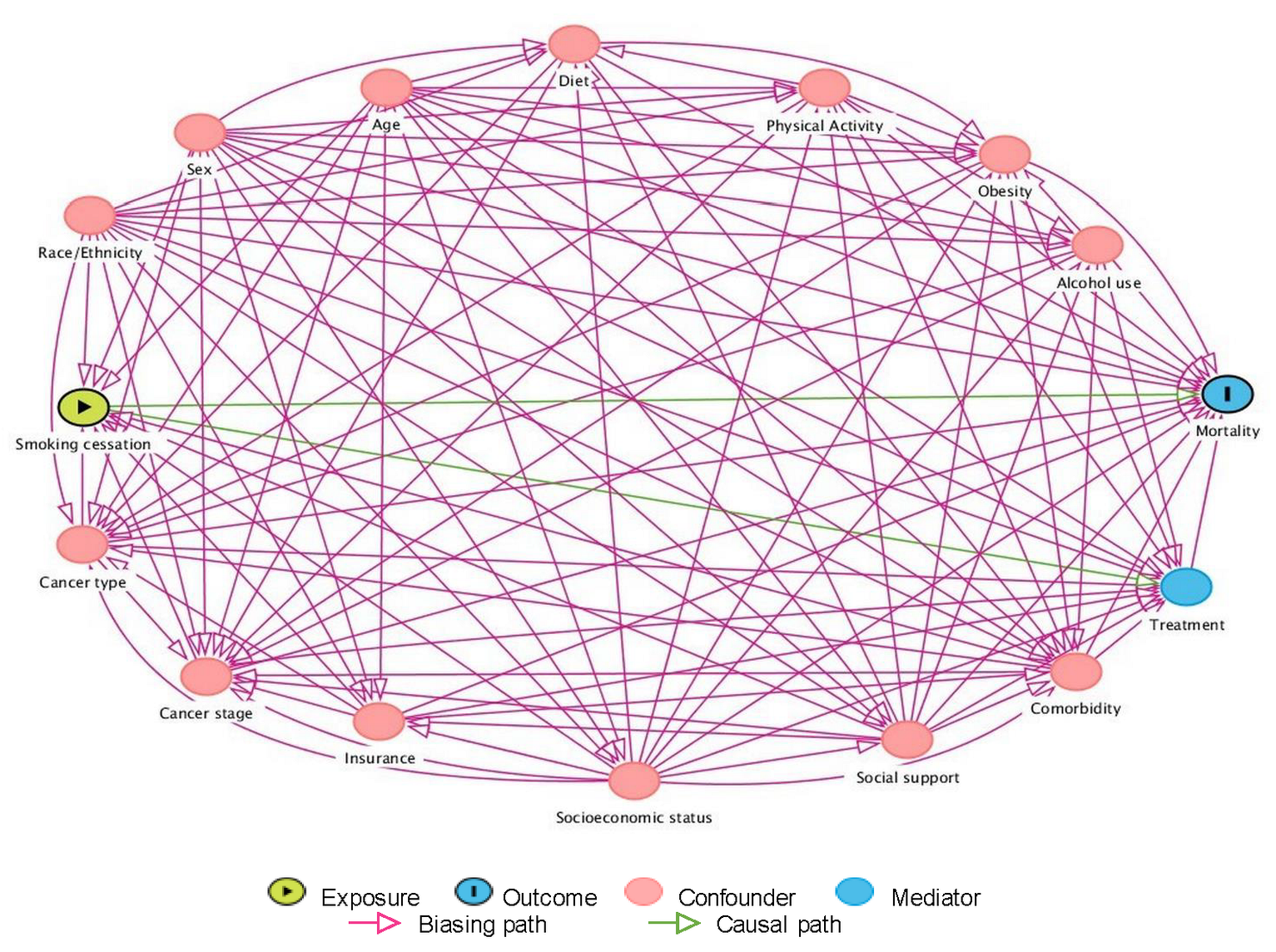
**

**Supplementary Table S2. Distribution of observations related to eligibility changes across sequential trials.**

|  | Trial 1 | Trial 2 | Trial 3 | Trial 4 | Trial 5 | Trial 6 | Total |
| --- | --- | --- | --- | --- | --- | --- | --- |
| Total observations | 369 | 331 | 285 | 254 | 219 | 196 | 1,654 |
| Initiated smoking cessation | 16 | 41 | 22 | 24 | 6 | 7 | 116 |
| Did not initiate smoking cessation | 353 | 290 | 263 | 230 | 213 | 189 | 1,538 |
| Mortality by the end of trial interval | 10 | 4 | 4 | 4 | 3 | ^a^ | 25 |
| Censored by the end of trial interval^b^ | 12 | 1 | 5 | 7 | 14 | ^a^ | 39 |

^a^Not relevant to the last trial

^b^Loss to follow-up or administrative censoring
